# Shoulder rhythm in patients with impingement and in controls: Dynamic RSA during active and passive abduction

**DOI:** 10.3109/17453670903153543

**Published:** 2009-08-01

**Authors:** Erling Hallström, Johan Kärrholm

**Affiliations:** ^1^Department of Orthopaedics, Uddevalla HospitalUddevallaSweden; ^2^Department of Orthopaedics, Institute of Surgical Sciences, Sahlgrenska University HospitalGothenburgSweden

## Abstract

**Background and purpose** Impingement syndrome is probably the most common cause of shoulder pain. Abnormal abduction and proximal humeral translation are associated with this condition. We evaluated whether the relative distribution between glenohumeral and scapular-trunk motions (the scapulohumeral rhythm) and the speed of motion of the arm differed between patients with impingement and a control group without shoulder symptoms.

**Patients and methods** 30 patients with shoulder impingement (Neer stage 2) and 11 controls were studied during active abduction and 21 patients and 9 controls were studied during passive abduction. Dynamic RSA at a speed of 2 simultaneous exposures per second was used to record the shoulder motions for 5–6 seconds.

**Results** Within the interval statistically evaluated (observations between 20–55° of relative active abduction in the glenohumeral joint), the patient group showed more scapular and trunk motions (p = 0.04), especially at up to 40°. The pattern of motion at passive abduction was somewhat similar to that in the controls.

Both controls and patients showed an increasing absolute (i.e. global) proximal displacement of the center of the humeral head with increasing active and passive abduction of the glenohumeral joint and humerus, without any certain difference between the groups. The mean maximum absolute proximal displacement in the patient and control groups amounted to about 30 mm and 20 mm, respectively. The corresponding relative displacement (with fixed scapula) was only 2.0 and 0.5 mm.

Active abduction was initiated with angular velocity of about 50 and 80 degrees per second, respectively, in the patients and the controls. In both groups it decreased with progressing abduction down to about 20 degrees per second (controls) after 3 seconds without there being any statistically significant difference. The angular velocities at passive abduction showed a similar pattern, still without any difference.

In both groups, the speed of proximal translation during active abduction peaked 0.5–1 second later than the speed of rotation and remained relatively even for about 1 second, followed by a deceleration.

**Interpretation** We found that the glenohumeral-thoracoscapular ratio during abduction of the arm in our study, measured as the distribution of motion between the glenohumeral joint and the trunk in both controls and patients with impingement, was less than or equal to 1:1. This finding differs from earlier results, probably due to the use of a method with high resolution and small influence of motions out of the frontal plane. The reason for reduced glenohumeral motions in the early phase of active abduction in the patient group is uncertain, but pain or avoidance of pain elicited by the motion was probably of importance.

## Introduction

The concept of shoulder subacromial impingement syndrome was introduced by Neer (1972). It corresponds to mechanical compression of the rotator cuff subacromial bursa and biceps tendon against the anterior undersurface of the acromion and coracoacromial ligament especially during elevation of the arm. Neer stated that as many as 95% of all rotator cuff tears could be attributed to mechanical impingement.

This theory was questioned by Budoff et al. (1998) who thought that 90–95% of all rotator cuff abnormalities could be attributed to intrinsic breakdown of the rotator cuff tendon because of tension overload overuse and traumatic injury rather than mechanical compression. Although controversial most authors acknowledge that compression is one of the factors that can result in rotator cuff pathology (Bigliani and Levine 1997, Michener et al. 2003).

Impingement is believed by many to be the most common cause of shoulder pain and it accounts for half of all patients who consult a physician because of shoulder pain (van der Windt et al. 1995, 1996, Vecchio et al. 1995). Working with the hands at or above shoulder level (Bjelle et al. 1981) has been found to be an occupational risk factor. During abduction of the arm the scapula rotates resulting in movement in the sternoclavicular and acromiclavicular joints—which tilts the glenoid fossa upward. This complex movement of the humerus scapula and clavicle is called the scapulohumeral rhythm (SHR) (Saha 1961, Freedman and Munro 1966, Doody et al. 1970, Poppen and Walker 1976).

Knowledge of the relative contribution of the glenohumeral joint to the total mobility of the arm in patients with impingement and healthy individuals is necessary in order to investigate the etiology of shoulder pain further. Conventional radiography is a well-known method for quantitative testing of shoulder mobility (Deutsch et al. 1996, Howell et al. 1988, Poppen and Walker 1976). This method has its limitations, however, because of projection artifacts and measurements in two dimensions.

Open MRI allows the shoulder to be studied with a varied positioning of the arm (Graichen et al. 1999, 2000, 2001) but the method does not permit dynamic analysis of shoulder kinematics.

In two previous studies we used dynamic radiostereometry to study the relative glenohumoral motions during active and passive abduction of the arm (Hallström and Kärrholm 2006, 2008). In the present study we evaluated the relative contribution of the glenohumeral joint to the global or total abduction of the arm including scapular motions and any motions of the upper trunk or body.

We focused on 3 questions: (1) What is the relative contribution of glenohumeral motion to the total or absolute active and passive abduction of the humerus? (2) Does the sequence of relative glenohumeral and other motions of the trunk differ between patients with impingement syndromes and the control group during abduction of the arm? (3) Is the speed of motion (angular velocity, velocity of proximal translation of the center of the humeral head) different in these groups?

## Patients and methods

Patients were recruited from a study on 3 treatment options for impingement. When asked to participate the patients could enter the clinical part of the study only or they could also undergo evaluation of shoulder motions using dynamic RSA (ethical committee consent R 475-1995-12-20). Controls were recruited from the working staff at orthopedic departments of Sahlgrenska hospital and Uddevalla hospital (ethics committee consent R 520-1998-08-20).

In the studies on the absolute active abduction 30 patients (mean age 50 (29–63) years, 20 men) who had been suffering from impingement (Neer stage 2) participated. They had had symptoms for at least 18 months. All patients were examined with ultrasonography. Exclusion criteria were presence of rotator cuff tear osteoarthritis and generalized joint disease such as rheumatoid arthritis. The corresponding control group consisted of 4 men and 7 women (mean age 38 (22–58) years) without shoulder symptoms.

In the studies on absolute passive abduction 21 patients (mean age 51 (29–63) years, 13 men) participated all of whom had had symptoms from impingement (Neer stage 2) for more than 18 months. Exclusion criteria were the same as for the previous group. The corresponding control group consisted of 4 men and 5 women (mean age 35 (22–58) years) without shoulder symptoms.

The sex distribution between the control groups and patient groups were not statistically significantly different in the studies on active and passive abduction (p = 0.09 and p = 0.28, respectively; Fisher's exact test) but the individuals in the control groups were younger (p = 0.008 and p = 0.004; Mann-Whitney test).

**Figure 1.**
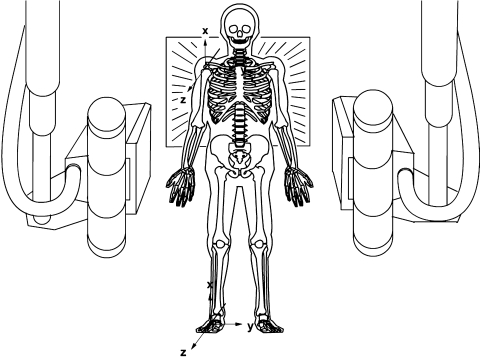
Reference position. The global coordinate system is fixed to the cage (illustrated here at floor level). At the reference examination the two body-fixed coordinate systems (one scapular one humeral) are aligned to the cage system.

4–6 spherical tantalum markers (0.8 or 1.0 mm in diameter) were inserted under local anesthesia into the scapula (acromion) and the humeral head. A set-up involving 2 film exchangers placed side by side (Figure 1) and designed for simultaneous exposure was used.

**Figure 2.**
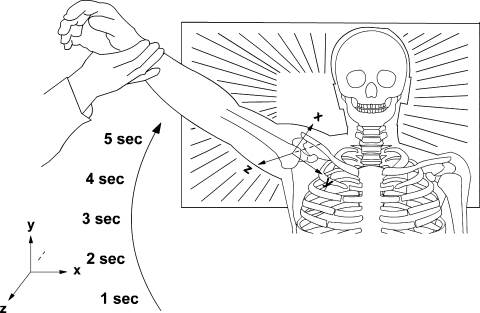
During motion the two body-fixed coordinate systems follow the motion of the bones which is illustrated here with changed position of the humeral coordinate system.

The vertical position of the film exchangers could be adjusted depending on the height of the patient. In front of the film exchangers a uniplanar calibration cage designed to suit the 2 film switchers was constructed and fixed. The exposure rate was set at 2 per second. 2–6 weeks after insertion of the one markers and using radiostereometric analysis (RSA Biomedical, Umeå, Sweden) the patients were studied standing during continuous active abduction and passive abduction (Figure 2) with the arm internally rotated. Because this recording technique limits the number of shoulder positions that can be studied, we chose to evaluate passive and active abduction used at an ordinary examination of the shoulder joint.

Together with one of the authors (EH) each subject (both patients and controls) performed several exercises of active abduction and passive abduction in order to feel as comfortable as possible before the radiographic examinations were started. The purpose was also to obtain as constant a speed as possible and to maintain the glenohumeral joint within the limits of the aperture (film size 35 × 35 cm).

When performing these exercises, the examiner stabilized the scapula with his hand until the patients themselves could maintain the scapula at as fixed a position as possible without any interference from the examiner. In this way we reduced the risk of additional body movements that could position the shoulder out of the field of view and we also avoided inclusion of any part of the examiner in the radiographic field of view during the recordings. Despite this, some of the examinations failed because the shoulder joint was not adequately visualized or the arch of motions was too small due to poor synchronization between the film exchangers and the patient.

The radiographic examination was initiated with a starting or reference position. A pair of stereo radiographs was taken corresponding to a well-defined anatomical position with the arm aligned to the longitudinal axis of the body and the forearm in external rotation with the palm facing forward. All subsequent recordings were related to this position of the arm.

The dynamic recordings lasted for 5–6 seconds (10–12 exposures). In the active abduction group only an average of 7 (5–10) representative pairs of stereographs (films) could be included in the final analysis in each patient due to difficulty in obtaining exact synchronization between the speed of the film exchanger and the motion of the arm. In the control group an average of 8 (7–9) film pairs were obtained. During passive abduction the corresponding values of representative pairs of stereographs (films) were mean 8 (6–10) and mean 8 (6–10), respectively.

A fictive point corresponding to the center of the humeral head was constructed to enable measurements of humeral head translations in a reproducible way. Circular templates were used to find the center of the head, but only on the 2 images of the reference position.

The radiographic films were scanned at 300 dpi using a flat-bed scanner (Sharp JX610, Osaka, Japan) and measured using dedicated software (Hallström and Kärrholm 2006).

Using the RSA digital software the positions of the centers in each of the shoulders were measured on the 2 images and their 3-dimensional coordinates were computed in the same way as for a tantalum marker. Thus this plotting was done once for each shoulder. Thereafter the position of this point was transferred to all other subsequent examinations of the same shoulder, using its computed position relative to the humeral head markers. The presence of documented stable and sufficiently well-scattered tantalum markers in the humeral head is a prerequisite for these computations (Nilsson et al. 1990).

**Figure 3.**
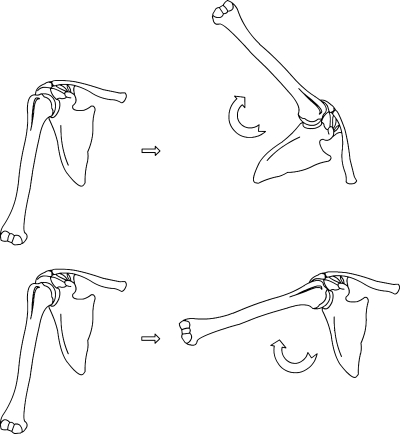
Simplified sketch to illustrate absolute or global motions (top) and relative humeral motions (bottom). Absolute humeral motions include changes of position caused by scapular and trunk motions whereas relative motions do not.

We measured the relative rotations and translations of the humeral head by using the scapula as a fixed reference segment. In RSA, this is done by computation of the absolute motions of the individual bones (scapula and humerus) in the global coordinate system defined by the cage. Thereafter a reversed matrix calculation is used to “replace” the scapula to its original position. The humeral segment defined by its markers is subjected mathematically to the same inverse rotation matrix (Figure 3). This enables computation of the relative difference between the two bones (segments) thus making it possible to evaluate motion occurring solely in one specific joint (here, the glenohumeral joint). In this study we also accounted for the computed humeral motions when related to the fixed cage coordinate systems. When these global motions (in RSA terminology, absolute motions) are computed the relative distribution of movement between the different parts of the body is disregarded. Thus the absolute motions of the humerus are the sum of any bending of the vertebral column movements of the chest, the scapula, and the humerus (Selvik 1974). The absolute motions are an objective recording of what the examiner actually can observe whereas the relative motions may be more or less accurately estimated by the clinical examiner based on his or her observations of the position of visual or palpable anatomical landmarks.

In RSA rotations are calculated in a specific order: first, around the transverse; thereafter, around the longitudinal, and finally around the anterior-posterior axis. When the rotations recorded are pronounced as in our study this order of calculation will have an influence on the interpretation of the results. The reason for this is related to the fact that the coordinate system follows the moving segment (here, the humerus). Since the patient and the examiner abducted the arm corresponding to rotations around the anterior-posterior (AP) axis we decided to adjust the position of the cage coordinate system 90° by rotation around the longitudinal axis. This means that in this study, rotations were calculated in the order abduction/adduction (AP axis) internal/external rotation (longitudinal axis), and flexion/extension (transverse axis).

To estimate the contribution of humeral abduction that did not occur in the glenohumeral joint we also recorded the absolute abduction of the humerus. These data were extracted from the same recording as that used to obtain information about the relative motions. The absolute abduction is the rotation of the humerus around the anterior-posterior axis in relation to the cage coordinate system. It is the sum of the relative abduction in the glenohumeral joint, the rotation of the scapula, and of the trunk.

In terms of mean error of rigid body fitting, marker stability measurements in the active abduction group were concerning the reference segment (scapulae), 0.099 (0.012–0.350) mm (SD 0.070), and 0.084 (0.016–0.334) mm (SD 0.052) in the moving segment (humerus). Qualitative analysis of marker scatter (mean condition numbers) in the scapula were 175 (72–455) (SD 82), and 129 (46–313) (SD 65) in the humerus. In the evaluation of the passive abduction the corresponding marker stability parameters were 0.094 (0.015–0.303) (SD 0.06) and 0.083 (0.01–0.334) mm (SD 0.051). The corresponding qualitative analysis of marker scatter gave mean condition numbers of 182 (73–592) (SD 97) and 130 (47–356) (SD 70). In this study we accepted high condition numbers provided there was high marker stability (a mean error of less than 0.050) in a series of examinations.

The reproducibility of the active abduction movement (6 patients) and passive abduction movement (2 patients) was tested by repetitions of the mobility of the arm after an interval of 15 min. Data for each type of motion analyzed were interpolated linearly at 5-degree intervals of active abduction/passive abduction.

Of the 52 patients selected for active abduction and 33 patients selected for passive abduction, 2 chose not to take part in the RSA evaluation after the randomization. 3 patients had a late diagnosis of cuff rupture and 7 patients with too poor a marker scatter in either of the bones were also excluded.

### Statistics

Statistical analyses using SPSS version 13.0 were based on recordings between 20° and 55° of relative active and passive abduction in the glenohumeral joint using scapulae as a fixed reference segment. This interval was chosen to maximize the number of observations available from each group.

Non-parametric tests were used in evaluation where each patient contributed with one observation. Repeated measures ANOVA (MANOVA) was used when each subject contributed when a series of dependent observations was made on the same subject. Non-parametric correlation was used. The significance level was set at p < 0.05. The test of repeatability pooled standard deviations was reported (a way of averaging) standard deviations are presented as a simplification to account for changes of variations during the arc of motion in each individual.

## Results

### Rotations around the anterior posterior axis—the scapulohumeral rhythm

In the control group the distribution of motion between the glenohumeral joint and the rest of the body was rather similar during both active and absolute passive abduction (Tables 1 and 2 and Figures 4 and 5; see also Supplementary data). At 20˚ of abduction in the glenohumeral joint this motion constituted about 40% of total (absolute) motion of the arm. With increasing abduction the maximum contribution amounted to as much as 50%.

**Table 1.** Combined spine, trunk, scapular and humeral motions (absolute motions) at increasing degrees of motions inside the glenohumeral joint (relative motion). Recorded values and distribution between the absolute and relative glenohumeral motions in percent are presented at active abduction

Patients – active abduction						Controls – active abduction					
Gleno- humeral mean **^a^**	Spine, trunk, scapula and glenohumeral		Gleno- humeral mean **^b^**	Spine, trunk, scapula and glenohumeral		Gleno- humeral mean **^a^**	Spine, trunk, scapula and glenohumeral		Gleno- humeral mean **^b^**	Spine, trunk, scapula and glenohumeral	
mean **^a^**	CI-95% **^a^**	mean **^b^**	CI-95% **^b^**	mean **^a^**	CI-95% **^a^**	mean **^b^**	CI-95% **^b^**
20	82	70–94	28	72	68–76	20	51	36–66	47	53	40–66
25	89	78–100	31	69	66–72	25	63	48–77	46	54	43–65
30	94	85–103	34	66	63–69	30	73	58–88	45	55	45–65
35	100	91–109	38	62	49–66	35	82	67–97	46	54	45–63
40	103	95–111	38	62	59–66	40	90	77–103	48	52	44–60
45	107	100–114	44	56	53–58	45	94	82–106	50	50	43–57
50	110	103–117	48	52	49–55	50	99	88–110	52	48	42–54
55	114	107–121	50	50	47–53	55	104	94–114	54	46	40–52
65	125	120–131	53	47	43–51	73	125	118–132	58	42	37–47
70	144	138–150	51	49	48–54	77	146	139–153	53	47	43–51

**^a^** degrees

**^b^** percent

**Table:** Tabel 2. Combined spine, trunk, scapular and humeral motions (absolute motions) at increasing degrees of motions inside the glenohumeral joint (relative motions) Recorded values and distribution between the absolute and relative glenohumeral motions in percent are presented at passive abduction

Patients – passive abduction						Controls – passive abduction					
Gleno- humeral mean **^a^**	Spine, trunk, scapula and glenohumeral		Gleno- humeral mean **^b^**	Spine, trunk, scapula and glenohumeral		Gleno- humeral mean **^a^**	Spine, trunk, scapula and glenohumeral		Gleno- humeral mean **^b^**	Spine, trunk, scapula and glenohumeral	
mean **^a^**	CI-95% **^a^**	mean **^b^**	CI-95% **^b^**	mean **^a^**	CI-95% **^a^**	mean **^b^**	CI-95% **^b^**
20	53	43–63	43	57	51–63	20	47	32–62	52	48	32–63
25	62	51–73	45	55	49–61	25	57	43–71	50	50	38–62
30	71	62–81	46	54	48–60	30	67	54–80	49	51	41–62
35	78	67–89	49	51	44–58	35	77	64–90	48	52	44–60
40	88	78–98	49	51	46–50	40	87	75–101	49	51	43–59
45	97	87–107	50	50	45–55	45	97	83–111	48	52	45–59
50	106	88–106	49	51	47–55	50	105	93–117	49	51	45–57
55	115	105–125	50	50	45–55	55	112	101–129	51	49	41–57
62	129	122–136	48	52	48–56	63	129	115–143	49	51	46–56
67	153	150–159	44	56	53–59	69	159	154–164	44	56	52–62

**^a^** degrees

**^b^** percent

The pattern of mobility at absolute passive abduction was rather similar in patients and controls (Table 2) (p = 0.8).

During active abduction the patients had reduced mobility in the glenohumeral joint up to 40˚ of relative abduction in the shoulder joint (Table 1). Thereafter the relative contribution of the glenohumeral joint was about the same as in the controls (all observations from 20 to 55˚ of relative glenohumeral abduction; p = 0.04).

### Proximal translation of the center of the head

Both the patient and control groups showed an increasing proximal displacement of the center of the humeral head with increasing passive and active abduction of the glenohumeral joint and humerus (Tables 3 and 4 and Figures 6 and 7; see supplementary data) without any statistically significant differences between the groups (absolute active abduction: p = 0.2; absolute passive abduction: p = 0.1). In the control group the mean maximum absolute proximal displacement amounted to about 20 mm and in the patient group it was about 30 mm. The corresponding relative displacement (with fixed scapula) was only 0.5 mm and 2 mm respectively.

**Table:** Tabel 3. Proximal translation of the humeral head in combination with active spine, trunk, scapular and humeral motions (absolute motion) at increasing degrees of motion inside the glenohumeral joint (relative motion). Recorded values in mm proximal translations of the humeral head are presented

Patients – active abduction						Controls – active abduction					
Relativ GH motion	Absolute motion	Relative proximal translation		Absolute proximal translation		Relative GH motion	Absolute motion	Relative proximal translation		Absolute proximal translation	
mean **^a^**	mean **^a^**	mean **^b^**	CI-95% **^b^**	mean **^b^**	CI-95% **^b^**	mean **^a^**	mean **^a^**	mean **^b^**	CI-95% **^b^**	mean **^b^**	CI-95% **^b^**
20	82	2.1	1.0–3.2	11.1	3.4–18.7	20	51	1.3	0.6–2.0	2.7	-3.2–8.6
25	89	2.3	1.0–3.6	14.0	6.1–21.9	25	63	1.3	0.2–1.7	4.8	-1.5–11.1
30	94	2.4	1.7–3.0	17.0	8.7–25.6	30	73	1.2	0.4–1.5	7.7	-0.9–14.5
35	100	2.4	1.6–3.2	20.6	12.3–28.8	35	82	1.0	0.1–1.9	10.6	2.4–17.6
40	103	2.4	1.6–3.2	24.0	15.7–32.1	40	90	1.0	-0.1–1.9	13.3	5.2–21.4
45	107	2.4	1.5–3.3	27.6	18.8–36.5	45	94	0.8	-0.3–1.8	16.3	7.6–24.4
50	110	2.2	1.3–3.2	31.2	22.1–40.2	50	99	0.6	-0.5–1.8	19.5	10.9–28.1
55	109	2.1	1.2–3.1	34.5	25.8–43.3	55	104	0.5	-0.8–1.7	23.0	14.1–31.9

**^a^** degrees

**^b^** mm

**Table:** Tabel 4. Proximal translation of the humeral head in combination with passive spine, trunk, scapular and humeral motions (absolute motion) at increasing degrees of motion inside the glenohumeral joint (relative motion). Recorded values in mm proximal translations of the humeral head are presented. At 55 degrees of relative motion there are missing observation at absolut proximal translations in patients and the control group.

Patients – passive abduction						Controls – passive abduction					
Relativ GH motion	Absolute motion	Relative proximal translation		Absolute proximal translation		Relative GH motion	Absolute motion	Relative proximal translation		Absolute proximal translation	
mean **^a^**	mean **^a^**	mean **^b^**	CI-95% **^b^**	mean **^b^**	CI-95% **^b^**	mean **^a^**	mean **^a^**	mean **^b^**	CI-95% **^b^**	mean **^b^**	CI-95% **^b^**
20	53	1.5	0.9–2.1	6.4	-0.2–13.0	20	47	1.1	0.7–1.9	-3.4	-18.0–11.4
25	62	1.7	1.1–2.3	8.7	2.0–15.3	25	57	1.0	0.3–1.6	-1.4	-13.4–16.1
30	71	1.8	0.5–3.0	12.0	5.0–18.9	30	67	0.9	0.1–1.8	1.3	13.3–15.9
35	78	1.6	0.3–3.0	15.9	8.1–23.6	35	77	0.9	-0.1–1.9	4.7	10.0–19.4
40	88	1.7	0.4–3.1	20.2	12.8–27.6	40	87	0.7	-0.5–1.8	8.9	-5.2–23.1
45	100	1.4	-0.1–2.8	24.7	16.9–32.4	45	97	0.6	-0.6–1.7	14.2	1.0–27.3
50	106	1.3	-0.3–3.0	30.6	23.4–37.9	50	105	0.2	-1.3–1.4	18.4	3.8–32.9
55	115	1.0	-1.0–3.1	36.1	28.7–43.5	55	111	0	-1.3–1.4	22.6	7.1–38.2

**^a^** degrees

**^b^** mm

### Influence of age

In the total material (patients and controls with 20–55º of relative abduction) there was no correlation between any of the variables recorded to describe shoulder motion and age (r = -0.1–0.2; p > 0.4). A separate analysis only including controls showed that the amount of active absolute shoulder rotation increased with decreasing age (relative abduction 40–55º: r = 0.64–0.66; p = 0.04). The other parameters studied did not show any correlation to age (r = -0.3–0.6; p > 0.05).

### Speed of motion

**Figure 8.**
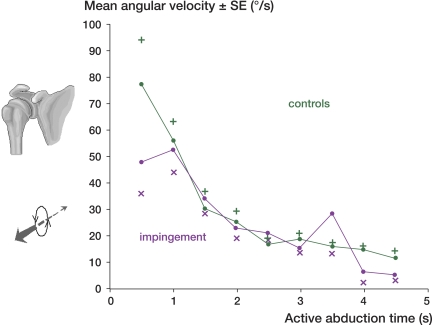
Angular velocity during active abduction. Patients with impingement versus the control group. Mean SE (p = 0.4).

Active abduction was initiated with angular velocity of almost 80 degrees per second in the controls and 50 degrees per second in the patients. It decreased with increasing abduction in both groups down to about 20 degrees per second (controls) after 3 seconds without any statistically significant difference (p = 0.4) (Figure 8).

**Figure 9.**
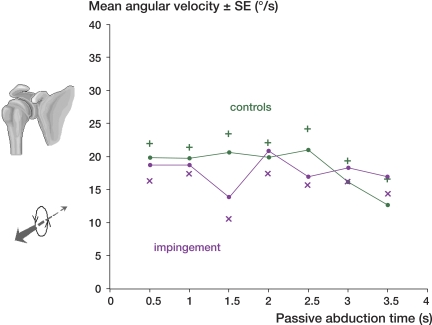
Angular velocity during passive abduction. Patients with impingement versus the control group. Mean SE (p = 0.7).

The examiner accelerated the passive abduction of the arm for 2 seconds up to about 40–50 degrees per second followed by a decelerating motion. As expected the speed of motion between the groups was rather similar (p = 0.7) (Figure 9).

**Figure 10.**
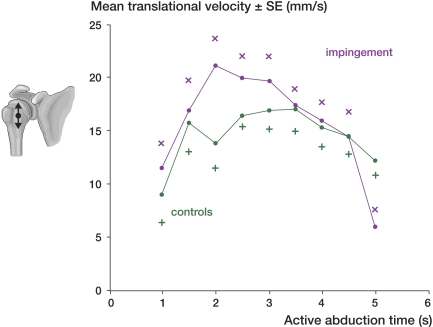
Translation velocity during active abduction. Patients with impingement versus the control group. Mean SE (p = 0.4).

**Figure 11.**
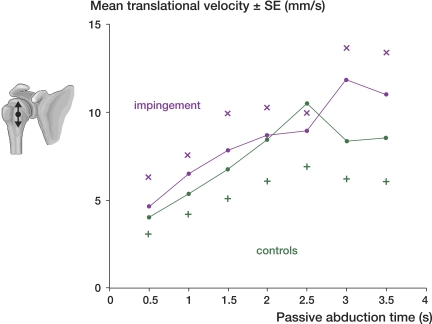
Translation velocity during passive abduction. Patients with impingement versus the control group. Mean SE (p = 0.5).

The speed of proximal translation during active abduction peaked 0.5–1 second earlier than the speed of proximal translation during passive abduction and maintained a more even speed of motion for about 1 second followed by a deceleration. There was no significant difference between patients with impingement and controls (p = 0.4) (Figure 10). The speed and pattern of translation during passive abduction corresponded to the pattern observed during active abduction and there was no significant difference between the patients and controls (p = 0.5) (Figure 11).

### Reliability

Repeated active abduction of the arm by the same examiner was associated with a variability corresponding to 4° (2 pooled SD) for absolute abduction. The corresponding value for absolute active proximal translation was 3.5 mm. The equivalent values for repeated passive abduction of the arm by the same examiner were 9° and 7 mm (2 pooled SD) for absolute abduction.

## Discussion

In this study, we did not evaluate the error of the RSA method itself because an absolutely fixed position of the shoulder joint could not be obtained which is necessary to study the actual marker configurations. Based on the documented marker stability (mean error of rigid body fitting) and marker scatter (condition number), 2 standard deviations of error for the motion parameters studied (proximal-distal translations and abduction-adduction) would most probably amount to 0.25 mm and 1 degree or less respectively. This would mean that most of the variability observed could be attributed to difficulties for the patient in repeating the same shoulder motion twice in a consistent way despite a preceding period of training. They could, however, do so with a higher reproducibility than could the examiner when performing the passive elevation. Thus, the variability presented is partly caused by technical considerations and partly by biological considerations.

Degenerative changes of the rotator cuff are thought to increase with age (Rathbun and Macnab 1970). In our study, we found no such correlation in the total material. In the control group the subjects were however more mobile when performing an active abduction regarding rotations around the anterior-posterior axes. This finding is difficult to interpret, not least because comparison of this parameter between patients and controls revealed no statistically significant difference. Concerning proximal translation during active abduction the only parameter that differed significantly between the groups we found no age-related influence.

In this study we isolated the relative abduction in the glenohumeral joint and related this mobility to the global motion of the arm, which is closer to what the examiner actually observes. This implies a simplification because out-of-plane movements are not accounted for and the contributions of different parts of the body to the absolute motions could not be mapped out in detail. For the purpose of our study we do, however, believe that analysis of shoulder abduction and proximal humeral translation is of particular interest. Recently we found that patients with impingement had significantly increased proximal translation of the humeral head at active relative abduction of the glenohumeral joint compared to a control group (Hallström and Kärrholm 2006). We also observed the same phenomenon when evaluating the Hawkins sign in patients with impingement (Hallström and Kärrholm 2008). Abnormal shoulder motions in these patients suggest that they also have reduced, delayed, or otherwise changed synchronization of motions in the glenohumeral joint.

We found that patients with impingement had a different distribution between absolute active abduction of the humerus and relative abduction in the glenohumeral joint. Even if the total amount of relative abduction was similar patients tended to reduce gleno-humeral abduction in the early phase of the motion. No such difference was observed during passive abduction. The reason for this difference is unknown, but it could be an effect of pain. Instead of using the glenohumeral joint which is probably more painful during the early phase of motion, patients activate their spinal and thoracoscapular muscles to benefit from bending of the spine and thereby reach the arc of motion, which is less painful. Another and perhaps less probable reason for early reduction of active glenohumeral abduction could be early degenerative changes in the acromioclavicular joint resulting in pain and secondary changes in the pattern of scapular and glenohumeral motions.

Freedman and Munroe (1966) and Doody (1970) analyzed abduction in the scapular plane on conventional radiographs. They computed a corresponding ratio of distribution between the glenohumeral joint and thoracoscapular joint to be 3:2, whereas Poppen and Walker (1976) measured a ratio of 5:4 after 30 degrees of abduction. In our study, the relative contribution of the glenohumeral joint to the absolute active or passive abduction was smaller. In both patients and controls it constituted only about 40% during the early phase and then gradually increased to around 50% during both passive and active motion. Thus the glenohumeral to scapula/trunk ratio was less than 1:1 during most of the observations.

The difference between our results and previously published observations may be related to the techniques used. Most of the early studies monitored scapulohumeral rhythm over 45-degree intervals. Greater variability was observed when measurements were taken at 30˚ increments. Inman et al. (1944) and Saha (1961) studied abduction in the coronal plane whereas others (Freedman et al. 1966 Doddy et al. 1970 Poppen et al. 1976) studied arm elevation in the scapular plane.

In a more recent analysis of arm elevation past 30° using an electromechanical device with a reported accuracy of about 1–2 mm and 1°, GH-to-ST ratios lower than 2:1 were found beyond 30°, which is more consistent with our findings (McQuade and Smidt 1998). Based on our results it does however seem that the scapular contribution to arm elevation is greater than previously reported.

Our analysis during passive abduction showed a similar pattern of distribution of glenohumeral and scapulothoracic motions in patients and controls. Grachien et al. (2001) could not find any difference in glenoid (scapula) rotation on sequential MRI images between 20 patients with impingement and 14 controls. They studied the shoulder at 30˚, 90˚, and 120˚ of abduction with and without muscle activity. These results are consistent with ours concerning the passive motion but differ concerning active abduction. This difference may be because the MRI studies were done statically whereas we exposed the shoulder joint during motion.

Grachien et al. (2000) studied the relative glenohumeral translation during active and passive abduction with 3-dimensional open MRI. 15 healthy subjects were studied at 5 different positions of passive abduction (30–150°) and at 3 different positions during active abduction of the shoulder with and without an adducting load to the arm at 60º, 90º, and 120º of adduction. The center of the glenoid and the midpoint of the humeral head were determined by 3D reconstruction and their relative position calculated. The authors found that the humeral head translated inferiorly 1–2 mm at both passive and active abduction with slightly reduced motions during muscular activity. Recently, we found that in a control group the center of the humeral head became displaced about 1 mm proximally during early passive and active abduction, and tended to be displaced slightly distally with proceeding abduction (Hallström and Kärrholm 2006, 2008). Even if the observations of Grachien et al. differed from ours performed during continuous shoulder motions the magnitudes of the displacement seen were within the range of 1–2 mm in both studies.

When we measured the absolute proximal/distal translation of the humeral head and included the entire shoulder girdle, we did not find that patients with impingement displaced their shoulders more proximally than normal during arm abduction. To our knowledge, there has been no previous study in the literature that supports or contradicts this observation.

As far as we know, the velocity of arm motions during active and passive abduction has not been studied previously either. We observed that during active motion of the arm, the peak velocity of the translation was reached 0.5–1 seconds later than that of passive abduction. We found previously that patients with impingement already in their early phase of active abduction displaced the humeral head center proximally relative to a fixed scapula and to a maximum level that was significantly more proximal than in the control group. This proximally displaced position was maintained throughout the abduction (Hallström and Kärrholm 2006).

To summarize, we found that the distribution of movement between the glenohumeral joint and the upper body including the scapula is less than or equal to 1:1 in both patients with impingement and those without any shoulder symptoms during both passive and active abduction which differs from earlier reports. Patients with impingement tended to reduce their glenohumeral abduction in the early phase of the motion. During abduction, the entire shoulder joint displaced 2–3 cm proximally. The relative displacement of the humeral head inside the shoulder joint was substantially smaller and constituted only up to 10% of this value (Hallström and Kärrholm 2006 2008). Reduced mobility of the glenohumeral joint during the early phase of active abduction may be an effect of pain or pain avoidance behavior. This pattern of reduced mobility was not found during passive abduction which supports the use of passive exercises in the early phase of rehabilitation.
